# A Review on Geopolymer Technology for Lunar Base Construction

**DOI:** 10.3390/ma15134516

**Published:** 2022-06-27

**Authors:** Sujeong Lee, Arie van Riessen

**Affiliations:** 1 Resources Utilization Division, Korea Institute of Geoscience and Mineral Resources, Daejeon 34132, Korea; crystal2@kigam.re.kr; 2 Resources Recycling, University of Science and Technology, Daejeon 34113, Korea; 3 John de Laeter Centre, Curtin University, Perth, WA 6845, Australia

**Keywords:** geopolymer, lunar base construction, lunar regolith, future work, ISRU

## Abstract

Geopolymer is a synthetic amorphous aluminosilicate material that can be used as an inorganic binder to replace ordinary Portland cement. Geopolymer is produced by mixing aluminosilicate source materials with alkali activators and curing the mixture either at ambient or low temperatures. Geopolymer research for lunar-based construction is actively underway to enable astronauts to stay on the moon for long periods. This research has been spurred on by earnest discussions of in situ resource utilization (ISRU). Recent research shows that the lunar regolith simulant-based geopolymers have high application potential to protect astronauts from the harsh moon environment. However, not all the simulants perfectly reproduce the lunar regolith, and the characteristics of the lunar regolith vary depending on the site. Issues remain regarding the applicability of geopolymer technology to contribute to ISRU through an elaborate and systematic plan of experiments. In this paper, the potential of geopolymers is assessed as a lunar-based construction material with the latest research results. Future work to develop the lunar regolith-based geopolymer technology is also proposed.

## 1. Introduction

Geopolymer is an amorphous aluminosilicate inorganic binder that can potentially replace ordinary Portland cement in concrete. The representative source materials for geopolymers are metakaolin and coal-fired fly ash. Geopolymers are produced by mixing an optimum amount of alkali activators with source materials and curing the mixture at ambient to elevated temperatures. In situ resource utilization (ISRU) on the moon is now being discussed in earnest, and research exploring the possibility of utilizing geopolymers as a building material for a lunar base for long-term stay astronauts is actively underway [1,2,3,4,5,6]. The catalyst for this research arises because the main component of the lunar regolith is aluminosilicate containing glassy phases ideal for reacting with alkali activators. Geopolymer technology has much to offer with advantages such as rapid strength gain, impressive fire resistance and notable thermal insulation performance. In most of the studies, lunar regolith simulants are used as the scarcity of actual lunar regolith prevents it from being used for experimentation. The extreme temperature of the lunar surface, with low gravity and near vacuum, raises considerable technological problems for the manufacturing of geopolymer. While the atmospheric environment in which charged dust particles are suspended is also significantly different from those on Earth and likely to create new technological problems such as electrical sparking and vacuum welding. Therefore, it is necessary to carefully examine whether it is possible to build a lunar base using geopolymer made from lunar regolith.

The lunar regolith is a mixture of unconsolidated material and rock debris covering the lunar surface [7]. In space, large and small meteoroids and charged particles from other planets and the sun constantly collide with the moon’s surface. The lunar regolith is formed by space weathering through sputtering and melting caused by solar wind and cosmic radiation. More than a third of the moon’s regolith is thought to contain glassy materials, which have the potential to be geopolymer precursors [8]. Due to the limited availability of actual lunar regolith, various simulants have been developed from rocks or volcanic ash on the Earth’s surface. Although these simulants have been designed to reproduce the mineral composition, particle size distribution and geotechnical properties of the lunar regolith, no simulant is identical to the lunar regolith. In addition, the lunar simulants are developed to address specific research purposes, such as resource extraction or to mimic local lunar regolith.

This review focuses on the assessment of the feasibility of geopolymer technologies for the construction of lunar bases by taking a general view of the mechanical properties, durability and cosmic radiation shielding performance of geopolymers manufactured from lunar regolith simulants based on the latest research results. Furthermore, the direction of future research and development, as well as the limitations of the research conducted to date, including technological uncertainties, are covered.

## 2. Applicability of Geopolymers as a Lunar Base Construction Material

### 2.1. Source Materials for Geopolymers

The most representative source material for geopolymers is metakaolin. When crystalline kaolin is heated to 700–800 °C for a few hours, dehydroxylation occurs, creating metakaolin (Al_2_Si_2_O_7_), an amorphous aluminosilicate. Since metakaolin is amorphous, it can be assumed that all of it is available as a precursor to producing geopolymer. When metakaolin, NaOH, silica fume and water are mixed in the ratio Na_2_O:Al_2_O_3_:4SiO_2_:11H_2_O [9], geopolymers with high compressive strength can be obtained. The Si/Al ratio of the geopolymer produced at this ratio is theoretically 2.0. In order to develop high strength in metakaolin-based geopolymers, the mixing ratio should be derived to achieve the Si/Al ratio in the range of 2 to 2.5 [10]. It is important to appreciate that even in ideal laboratory conditions, not all of the aluminosilicate precursors react, leaving a microstructure with geopolymer, unreacted metakaolin and pores [11].

Low Ca Class F fly ash is the second most used source material for geopolymers. Class F fly ash emitted by pulverized coal combustion (PCC) typically contains mullite that is crystallized at high temperatures plus quartz and iron oxides. However, the most important component of fly ash for making geopolymer is the amorphous material, with the amount and ratio of Al_2_O_3_ and SiO_2_ being paramount for activation by alkali. As every fly ash has a different level of amorphous content with varying Al_2_O_3_ and SiO_2_ ratios, it is essential to accurately determine these values to enable a geopolymer formulation to be calculated. Quantitative X-ray diffraction (QXRD) is the most direct and robust technique for determining the amount of amorphous material and its composition [11]. With the correct formulation and proper curing process, the compressive strength of fly ash-based geopolymers can reach values ≥100 MPa [11,12,13,14,15].

A third geopolymer precursor is a volcanic ash made up of rock and glassy substances ejected when a volcano erupts. Volcanic ash is largely divided into pumice and scoria according to its chemical composition and particle shape. Scoria contains crystalline minerals because it is formed from basaltic magma by slow cooling. Its SiO_2_ content is around 50%, and the color is dark. In many cases, scoria is used as source material for lunar regolith simulant because the basaltic chemical composition of scoria and the crystalline–glassy mixture properties are similar to lunar regolith (Table 1). Both pumice and scoria can be used as source materials for geopolymers, as they contain large amounts of reactive Al_2_O_3_ and SiO_2_, such as fly ash from thermal power plants.

### 2.2. Advantages of Geopolymers as Lunar Base Construction Materials

The potential for geopolymers as a lunar base construction material is best demonstrated by comparing them with ordinary Portland cement (OPC) (Table 2). If a properly formulated geopolymer mixture is cured below 100 °C, the ultimate strength of a low-Ca fly ash geopolymer can be obtained in 24 h. Ambient curing of geopolymer is usually achieved by adding low amounts of Ca with ultimate strength gained over 28 days. For OPC-based concrete compressive strength at 28 days is used as the design reference for ambient cured samples. 

**Figure 1:**
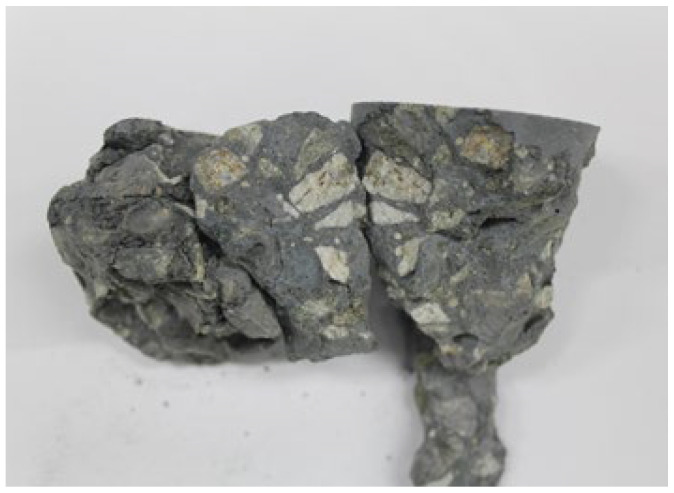
Fracture surface showing cracking through aggregate particles in geopolymer concrete.

Unlike the interfacial transition zone (ITZ) between cement and aggregate, which is the weakest part of ordinary Portland cement concrete [22], the ITZ in geopolymers is generally stronger [23]. For this reason, when a geopolymer concrete fractures, failure may occur through the aggregate rather than along the boundary between the geopolymer and the aggregate (Figure 1). 

In addition, geopolymers are resistant to acids [24], sulfates and chlorides, which cause cement concrete deterioration. Moreover, ASR (alkali–silica reaction), which causes cracking and deterioration of cement concrete, is less severe in geopolymer [27]. Above all, geopolymer is a material with excellent fire resistance that can withstand temperatures up to 900~1000 °C without spalling. Depending on the formulation, the compressive strength of some geopolymers was observed to increase at high temperatures [13,26]. The superior physical and chemical properties of geopolymer compared to OPC makes it a promising prospect to be manufactured from lunar regolith and is thus an ideal construction material for the purposes of ISRU.

A potential drawback of geopolymerization is the high viscosity of the alkalis and the subsequent paste, creating workability issues. NaOH-activated geopolymer paste is highly viscous, while KOH-activated geopolymer paste is much less viscous, albeit more expensive [27]. In order to improve the workability of viscous geopolymers, plasticizers used for the purpose of reducing the amount of mixing water required for OPC are also used in geopolymers but are not very effective [28,29]. In the case of metakaolin-based geopolymers, the use of methyl isobutyl carbinol (MIBC) has the effect of simultaneously improving flowability and strength [30].

## 3. Utilization of Lunar Regolith as a Raw Material for Geopolymers

### 3.1. Composition of Lunar Regolith and Its Simulants

The reason lunar regolith has excellent potential as a geopolymer precursor is its mineral composition. The major constituent minerals of the lunar regolith are olivine, pyroxene, plagioclase, ilmenite and silica (Table 3) [31,32,33]. The regolith thus consists of aluminates and/or silicates except for the ilmenite. The bulk composition of the lunar regolith is similar to the composition of the Earth’s crust, which is 40–50% SiO_2_ and 10–20% Al_2_O_3_. Collins et al. (2022) conducted a thorough characterization of a range of lunar simulants [34] and provided a summary of the amorphous content of regolith from the Apollo mission flights. The average amorphous content was found to be approximately 33 wt.%. In addition, more than one-third of the lunar regolith is made up of agglutinates and vitreous micro-spherules [31,35]. Because agglutinate can account for 60–70% of the lunar regolith [35], it is a highly promising source of reactive aluminosilicate source material for geopolymers.

Since the lunar regolith simulants reflect the chemical composition of the lunar regolith, the SiO_2_ and Al_2_O_3_ content are suitable for the production of geopolymer (Table 1). In addition, with the CaO content in the range of 6~10%, it is also suitable for geopolymerization (Table 1). Simulant JSC-1A is manufactured from the basalt of Merriam Crater and is similar to the regolith of the Mare area. Simulant BP-1 has a higher TiO_2_ content than the chemical composition of the lunar regolith since it was developed for geotechnical purposes. LHS-1 is a simulant that reproduces the chemical composition and particle size distribution of the regolith in the Highland area and has higher Al_2_O_3_ and CaO content compared to other stimulants. Among the simulants manufactured from Chinese volcanic ash (GVS, LN, BH-1, BH-2), BH-1 reproduces the particle size distribution of the Apollo 16 sample. DNA-1 is a simulant developed by the European Space Agency to reproduce the regolith of the lunar Mare area, consisting of 75% crystalline and 25% glassy particles.

### 3.2. Recycling of the Mixing Water

Water is not a component of geopolymer, with its role being to transport ions in the geopolymer mixture and enable the mixing of the ingredients. Essentially, water is necessary for the production of the geopolymer, but ideally, the mixing water can be recovered after the geopolymer has set and hardened. In 2018, NASA announced that a significant amount of ice water was present in craters on the lunar poles. This discovery brought a positive shift to the concept of ISRU on the moon. However, it would be very difficult to retrieve water from the polar craters due to the cryogenic temperatures experienced in this region of the moon. In 2020, observations from SOFIA, a joint observatory between NASA and the German Space Agency, were presented with clear evidence of water, for the first time, on the sunlit surface of the moon [36]. This positive discovery suggests that mixing water can be secured for the production of geopolymers in several areas on the moon.

As mentioned above, an advantage of geopolymers as a lunar base construction material is that the mixing water can be recycled. Wang et al. (2016) presented a sustainable model in which most of the water used is recovered and reused after the geopolymer is manufactured from tektites, a round gravel-sized material that has been melted by meteorite impact, ejected up into the atmosphere and then fallen back to Earth [5]. The main components of tektite used by Wang et al. (2016) are SiO_2_ 69.84% and Al_2_O_3_ 12.16%. In this study, the residual moisture content was found to be 0.8~1.77% in tektite-based geopolymers cured at 60 °C for one day and then heated in a vacuum at 120 °C for 8 h. In geopolymers, there is physically bound vaporizable moisture and chemically bound residual moisture. Although there is controversy about the form of chemical moisture, as present in Barbosa’s model [37], residual moisture is likely to exist in the Si-Al tetrahedral framework in the form of OH^−^ bound to cations or in hydrated Na ion clusters. The strength of tektite-based geopolymer was maintained or decreased by only about 10% even after 30 cycles of 30 min at −196 °C and 30 min at room temperature. The sustainable production of geopolymers from lunar regolith accomplished by recycling most of the mixing water, as proposed in Wang et al. (2016), is likely to be realized when the following two requirements are satisfied [5]. First, the lunar regolith as source material for geopolymers must be an aluminosilicate with the low calcium content. Many studies reported that hydrates such as C-A-S-H or C-S-H are formed in the reaction product in the presence of calcium [38,39,40]. The presence of hydrates, depending on their content, weakens the advantages of the geopolymer and reduces the amount of evaporable moisture that can be recovered. The second is to seal the curing space to prevent moisture from escaping during the initial curing and mixing of the geopolymer in the near-vacuum atmosphere of the moon. This would not be technically easy in the moon’s atmosphere, which is made up of a thick layer of suspended charged dust particles. 

## 4. High Potential of Lunar Regolith Simulant-Based Geopolymers

### 4.1. Selection of Alkali Activator

In general, geopolymers are prepared by activating aluminosilicate source materials with high pH alkali hydroxides or silicates. The most commonly used activators are alkali hydroxides, such as caustic soda (NaOH) or potassium hydroxide (KOH), with the former more widely used as it is less expensive. In most studies of lunar regolith simulant-based geopolymer, caustic soda solution was solely used (Table 4). Activators made from a combination of soda solution and sodium silicate or potassium silicate were also used. The advantages of NaOH are that some crystalline aluminosilicate minerals are generally more soluble in NaOH than in KOH [41], in addition to the more readily dissolvable amorphous material. The greater the degree of Si and Al dissolution, the greater the potential strength of the geopolymer. However, NaOH’s high viscosity and solubility decrease rapidly as the temperature reduces [42], suggesting that mixing and curing times need to be carefully selected. In the case of KOH, the viscosity is lower than that of NaOH, and geopolymer prepared with KOH may be preferred in that it exhibits lower thermal expansion [43]. However, since K is larger than Na, it is more exothermic when dissolved in water [44], which needs to be managed when processing geopolymer. Sodium silicate solution, or water glass, is not always used alone as an activator but may be combined with caustic soda so that targeted Si/Al and Na/Al values can be achieved in the geopolymer.

One-part geopolymer, instead of a conventional two-part geopolymer design, can be produced by adding free water to a mixture of powdered activator and geopolymer source material [45,46]. Solid sodium silicate, caustic soda powder, CaO, MgO, red mud, etc., may all be used as activators [43]. The mechanical strength of one-part geopolymers was found to be comparable to that of two-part geopolymers prepared with liquid activators [46]. On the moon, a one-part geopolymer manufacturing method would be more appropriate. It may be more suitable to use KOH rather than NaOH in that it can exhibit higher flowability during mixing and better heat resistance of the final geopolymer. Depending on the amount of reactive silica and alumina in the lunar regolith, optimization of mechanical properties, heat resistance and durability of the geopolymer will be possible by changing the targeted Si/Al ratio by selecting either sodium silicate or sodium aluminate powder as required.

### 4.2. Compressive Strength and Durability of Lunar Regolith Simulant-Based Geopolymers

It is advantageous to use a combination of sodium silicate solution and NaOH to achieve higher compressive strength, especially when targeting specific Si/Al values [50]. Since the gravitational force on the moon is only 1/6 of that of the Earth, approximately 6 MPa is sufficient compressive strength for lunar base construction, compared with 35 MPa required for a one-story structure on the Earth [4]. Based on this value, it is clear that sufficient compressive strength of lunar regolith simulant-based geopolymers can be obtained when cured at ambient in the air (Table 4) [1,3,4,6,49]. 

The compressive strength of lunar regolith simulant-based geopolymers was found to decrease by about 50% when cured in a vacuum compared to ambient curing [1]. On the other hand, geopolymers cured at slightly higher temperatures (30.7–99.6 °C) for 72 h were found to have higher strength when cured in a vacuum [3]. In addition, it was stated that curing in a vacuum is advantageous for strength development by preventing efflorescence [3]. Generally, rapid drying of geopolymer during curing causes microcracking, leading to strength loss. Pilehvar et al. (2021) reported that curing in a vacuum increases porosity and reduces strength, although the pressure (vacuum) used in Pilehvar’s experiment was many orders of magnitude greater than that experienced on the moon [51]. From these results, it was inferred that careful selection of the curing scheme is essential to maximize the strength of geopolymers manufactured on the moon, and importantly, the processing must also be practical on the lunar surface.

Pilehvar et al. (2020, 2021) found that when conducting durability tests, the compressive strength of geopolymers increased when repeatedly exposed to a low–high-temperature cycle similar to that experienced on the lunar surface [21,51]. The compressive strength of geopolymer was found to increase by a factor of 6 or more when exposed to a thermal cycle of −80~114 °C in air and vacuum. In another study by Xiong et al. (2022), geopolymer exposed to temperature cycles of −190~25 °C was found to have gained compressive strength while the compressive strength of samples exposed to 0~−30 °C cycles increased from 60 MPa to 80 MPa [18]. Xiong et al.’s explanation for the strength increase was that the moisture trapped in the pores was frozen, and since the strength of the ice was greater at lower temperatures, the ice in the pores contributed to the increase in the strength of the geopolymer [18]. Zhang et al. (2022), on the other hand, reported opposite results in that the compressive strength of geopolymers exposed to the lunar surface temperature range of −179.8~99.6 °C decreased [3]. According to mercury intrusion porosimetry (MIP) measurement, after exposing samples to lunar surface temperature cycles, the strength of geopolymers decreased by 15~18% due to the increase in porosity and cracking [3]. Even after the reduction in compressive strength, the residual strength was at least 13 MPa, which is still sufficient for the construction of a lunar base. The flexural strength, however, showed a larger decrease of 49~70%, compared with a mild decrease in compressive strength [3]. Although the results of changes in the compressive strength of geopolymers exposed to lunar surface temperature cycles are inconsistent, the research results overall are generally positive.

The cosmic radiation shielding effect has only been studied for geopolymers made from JSC-1A simulant (Table 4). By using a simulation program, geopolymer was found to have shielding performance adequate for a 12-month stay by humans on the moon, assuming no extreme solar flare events. The equivalent dose would be equal to 5 cSv, which is the annual whole-body radiation worker limit on Earth [4]. In another study by Ferrone et al. (2022), a regolith-binder thickness of 1 m would be required to reduce the galactic cosmic radiation (GCR) dose by half [47]. Ferrone et al. (2022) based their simulations on a 14-day lunar stay [47]. In these two studies, the geopolymers were prepared with a combined activator of caustic soda and water glass, but the Si/Al ratio of the geopolymers prepared by each team was not specified. As the most important factor influencing the properties of a geopolymer is the chemical composition of the geopolymer, it is necessary to evaluate the shielding properties of geopolymer with different Si/Al ratios. Much of the above discussion is based on geopolymer made from a simulant. Once successful in achieving adequate geopolymer binder strength, the potential of adding filler or aggregate using other lunar regolith minerals can be explored. Two things are achieved by doing this: first, a low binder:aggregate ratio means less binder is needed, and secondly, aggregate with high cosmic radiation absorption properties can be included.

**Figure 2:**
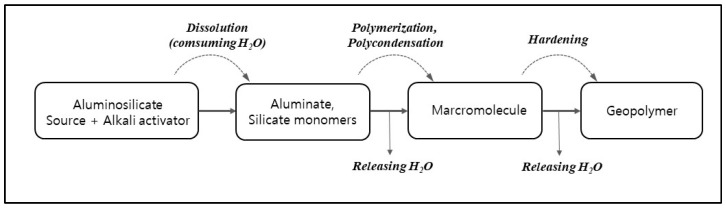
Geopolymerization reaction based on the conceptual model of Duxson et al. (2007) [53].

In order for more extensive dissolution during the early stages of geopolymerization, it is important to preserve moisture in the geopolymer mixture (Figure 2), as this is when water is consumed. The lunar surface temperature varies depending on the location, and it can reach up to 125 °C [52]. Temperatures above 50 °C last for about 9 days at 30° latitude on the moon. Compared to conventional thermal curing for 24 h on Earth, ambient curing provides sufficient strength on the moon. However, evaporation of moisture inhibits the initial geopolymer reaction and leads to a decrease in compressive strength. Therefore it is technically crucial to mix the source materials and seal the geopolymer mixture during curing.

## 5. Conclusions

Research results based on geopolymers made from lunar topsoil simulants are very positive and support the proposition that geopolymers could be used as a lunar base construction material. The geopolymer structures would protect long-stay astronauts from extreme temperature and cosmic radiation while achieving ISRU targets of the utilization of lunar regolith. However, given that any lunar regolith simulant does not perfectly reproduce the lunar regolith, and the characteristics of the lunar regolith vary depending on the landing site, issues remain to be solved for this applicability to be realized in the ISRU scope.

Ideally, in ISRU, it would be the use of a one-part geopolymer that is produced by adding only water rather than the conventional two-part geopolymers. For this to be achieved, the type and amount of the activator in powder form that is most suitable in terms of cost and performance of the geopolymer should be thoroughly evaluated. Above all, since most of the lunar regolith particles have an angular shape, ways to increase the workability of geopolymers on the moon and how to promote curing in a vacuum over a wide temperature range of −171 °C to 120 °C or higher will need to be carefully addressed in the future. Recycling the blended water is absolutely necessary, and the durability of geopolymers is expected to be improved if moisture is recovered. The most difficult problem is to develop a quantitative mixing method tailored to the characteristics of lunar regoliths, such as a method for calculating the mix proportions of fly ash-based geopolymers with high compressive strength.

## Figures and Tables

**Table 1:** Lunar regolith simulants used in the studies of manufacturing geopolymers for lunar base station. PSD = particle size distribution.

Simulant	Chemistry (wt.%)			Source	Note	Ref.
	SiO_2_	Al_2_O_3_	CaO			
JSC-1A	46.67	15.79	9.90	Basalt cinders from Merriam Crater	Similar to low-titanium lunar mare terrain, formulated to be close to JSC-1	[6,16]
BP-1	47.2	16.7	9.2	San Francisco Volcanic Field	Lack of chemical similarity to Apollo samples	[17]
LHS-1	48.1	25.8	18.4	Not sourced from any particular terrestrial source	High similarity to the highlands soil in terms of chemical composition and PSD	[1]
GVS	43.3	16.5	8.8	Volcanic scoria cones	Same origin of CAS-1 and NEU-1	[3]
LN	44.83	14.18	8.93	Volcanic scoria cones	Similar mineralogy to Apollo samples	[18]
BH-1	43.3	16.5	8.8	Volcanic scoria cones	Mineralogical and chemical analog to Apollo 16 samples	[19]
BH-2	43.3	16.5	8.8	Volcanic scoria cones	Upgraded to have the same gradation to Apollo 17 samples	[20]
DNA-1	47.79	19.16	8.28	Dini Engineering srl for Monolite UK Ltd.	Glass content of 25 vol%	[21]
LMS-1	42.81	14.13	5.94	Exolith Lab.	Lunar mare simulant	LMS-1 Fact Sheet, Exolith Lab, FL

**Table 2:** Comparison of geopolymer with OPC on Earth.

	Geopolymer	OPC
Advantages	Rapid strength gain Higher chloride resistance Acid and sulfate resistance [24,25] Excellent fire resistance [13,26] Impressive heat insulation Superior acid resistance [24] Frost resistance Little or no alkali–silica reaction [27] Strong ITZ [23]	Shorter setting time Faster hardening Ambient curing Vasts amounts of available resource
Disadvantages	Lower workability May need thermal curing Safety issues re: working with highly alkaline solutions	Higher drying shrinkage and cracking Lower durability High CO_2_ emission Alkali–silica reaction Weaker ITZ [22]

**Table 3:** Mineralogical properties of major and minor minerals present on the moon.

Mineral	Formula	Specific Gravity	Mohs Scale	Impurities
Major minerals				
olivine	(Mg, Fe)_2_SiO_4_	3.2–4.5	6.5–7	Mn, Ni
pyroxene	(Ca, Mg, Fe)_2_Si_2_O_6_	3.2–3.3 (enstatite)	5–6 (enstatite)	Mn, Li, Na, Al, Sc, Na, Ti, Co
plagioclase	Ca_2_Al_2_Si_2_O_8_	2.76 (anorthite)	6–6.5	
ilmenite	FeTiO_3_	4.7–4.8	5–6	Mn, Mg
silica	SiO_2_	2.2–2.6	7 (quartz)	Ti, Fe, Mn (quartz)
Minor minerals				
apatite	Ca_5_(PO_4_)(F, Cl)	3.2	5	REE ^#^
baddeleyite	ZrO_2_	5.5–6	6.5	Hf
chromite-ulvöspinel	FeCr_2_O_4_-Fe_2_TiO_4_	4.8–5	5.5–6	Al, V, Mn, Mg, Ca
iron	Fe(Ni, Co)	7.9		Ni, Co
merrillite *	(Ca_3_)(PO_4_)_2_	3.1		Mg, Na
pleonaste	(Fe, Mg)(Al, Cr)_2_O_4_	3.6–3.9	7.5–8	Mn
rutile	TiO_2_	4.2	6–6.5	Nb, Ta
feldspar	(Ca, Na, K)AlSi_3_O_8_	2.6	6–6.5	Rb, Ba
troilite ^#^	FeS	4.7–4.8		
zircon	ZrSiO_4_	4.6–4.7	7.5	
zirkelite-zirconolite	(Ca, Fe)(Zr, Y, Ti)_2_O_7_	4.7	5.5	Th, U, Ce, Nb
dysanalyte	(Ca, Fe)(Ti, REE)O_3_	4–4.3 (perovskite)	5–5.5 (perovskite)	
thorite	ThSiO_4_	6.6–7.2	4.5–5	U
titanite	CaTiSiO_5_	3.5–3.6	5–5.5	Fe, Al, REE, Th
tranquillityite *	Fe_8_(Zr, Y)Ti_3_Si_3_O_24_	4.7		Y, Al, Mn, Cr, Nb, REE
yittrobetafite *	(Ca, Y, U, Th, Pb, REE)_2_ (Ti, Nb)_2_O_7_			

* Extraterrestrial only; ^#^ REE = rare earth element.

**Table 4:** Comparison of properties reported for lunar regolish simulant-based geopolymers.

Source	Simulant	Activator	Curing Temperature	Compressive Strength (MPa)	Note
[1]	BP-1 JSC-1A LHS-1	SS	20 °C at 1 atm and vacuum for 7 d, followed by −80 to 600 °C curing	5–10 (20 °C at 1 atm) 18–35 (20 °C (1 d)→600 °C (1 h)) 1–4 (20 °C at 1 atm (7 d)→vacuum at 20 °C) Unconsolidated (20 °C (4 d)→−80 °C (3 d)	Reduced CS for GPs cured under vacuum and exposed to sub-zero temperatures, Positive effect of high amorphous Al-Si content and high proportion of fines
[2]	BH-1	NaOH	30.7→99.6→33.5 °C (discontinuous) for 24 and 72 h, followed the temperature variation cycle ranging from −178.9 °C to 99.6 °C	16–38 at different temperature regimes, 15–18% decrease in CS after the cycle, 49–70% decrease in FS after the cycle	Durability test (lunar surface high and cryogenic temperature variation cycle at 30° latitude). Noticeable degradation after the cryogenic attack with increased porosity
[3]	GVS	NaOH+SS	20, 40, 60, 80 °C at 1 atm	19 (20 °C), 42 (40 °C), 69 (60 °C), 76 (80 °C)—28 d	Curing temperature—the most significant factor influencing CS
[47]	JSC-1A	SS (s)	Mixing simulant with SS followed by calcining at 260 °C for 1 h and 127 °C in air and vacuum for 1 h	Rockwell Hardness of 75 (RH 80 for annealed titanium)	Adequate space radiation shielding of ‘Regishell’ (simulant + 10% SS binder) (by Monte Carlo simulations)
[18]	LN	NaOH+SS	60 °C for 7 d	59 (7 d) 50 at 120 °C 80 at −30 °C	Increased CS after 40 cycles of thermal shock (−196 °C for 1 h to 25 °C for 1 h)
[48]	DNA-1	NaOH	80 °C for 3 h, followed by a lunar day-and-night cycle at −80 to 114	1 (at 1 atm), 13 (after lunar cycle at 1 atm), 4 (after lunar cycle at vacuum)	Beneficial use of urea 3% Increased CS after LDN cycle Reduced CS by vacuum dehydration
[20]	BH-2	NaOH	30.7–99.6 °C at 1 atm and at vacuum for 0–72 h	19 (24 h), 38 (72 h) at vacuum 20 (24 h), 33 (72 h) at 1 atm	Cured under lunar surface T variation Higher CS under vacuum curing
[21]	DNA-1	NaOH	80 °C for 6 h, followed by freeze-thaw cycles at −80 to 80	16 (0 cycles), 25 (2 cycles), 24 (4 cycles), 32 (8 cycles)	Beneficial use of urea 3% for 3D printing, highest CS for pure GPs
[49]	JSC-1A	NaOH, NaOH+ K_2_SiO_3_	at RT for 28 d	2 (2 M NaOH)-18 (8 M NaOH)	Less reduction in flexural strength with respect to CS beneficial use of urea.
[6]	JSC-1A	NaOH+SS	26 °C at 1 atm 26 °C at vacuum 106 °C at 1 atm	10–12 (7 d) 11–12 (7 d), 9–10 (28 d) 9–13 (7), 10–20 (28 d)	Compression molding, 106 °C = average lunar daytime heat
[4]	JSC-1A	NaOH+SS	106 °C at vacuum 23 °C for 7 d 60 °C for 3 d (pouring and compression molding)	17 (3 d, conventional pouring) 38 (3 d, compression molding) 33 (7 d, compression molding)	Adequate radiation shielding and thermal insulation of ‘Lunamer’ (by FLUKA simulations)

Code: SS = sodium silicate; CS = compressive strength; GP = geopolymer; FS = flexual strength; RT = room temperature.

## Data Availability

Not applicable.
